# Chondrogenesis mediates progression of ankylosing spondylitis through heterotopic ossification

**DOI:** 10.1038/s41413-021-00140-6

**Published:** 2021-03-17

**Authors:** Tao Yu, Jianguo Zhang, Wei Zhu, Xiao Wang, Yun Bai, Bin Feng, Qianyu Zhuang, Chang Han, Shengru Wang, Qimiao Hu, Senbo An, Mei Wan, Shiwu Dong, Jianzhong Xu, Xisheng Weng, Xu Cao

**Affiliations:** 1 Department of Orthopedic Surgery, Johns Hopkins University School of Medicine, Baltimore, MD 21205 USA; 2 Department of Orthopedics, Southwest Hospital, Third Military Medical University, Chongqing, 400038 China; 3 Department of Orthopedics, Peking Union Medical College Hospital, Chinese Academy of Medical Sciences & Peking Union Medical College, 100730 Beijing, China; 4 State Key Laboratory of Complex Severe and Rare Diseases, Peking Union Medical College Hospital, Chinese Academy of Medical Science & Peking Union Medical College, 100730 Beijing, China

**Keywords:** Pathogenesis, Calcium and phosphate metabolic disorders

## Abstract

Ankylosing spondylitis (AS) is chronic inflammatory arthritis with a progressive fusion of axial joints. Anti-inflammatory treatments such as anti-TNF-α antibody therapy suppress inflammation but do not effectively halt the progression of spine fusion in AS patients. Here we report that the autoimmune inflammation of AS generates a microenvironment that promotes chondrogenesis in spine ligaments as the process of spine fusion. Chondrocyte differentiation was observed in the ligaments of patients with early-stage AS, and cartilage formation was followed by calcification. Moreover, a large number of giant osteoclasts were found in the inflammatory environment of ligaments and on bony surfaces of calcified cartilage. Resorption activity by these giant osteoclasts generated marrow with high levels of active TGF-β, which induced new bone formation in the ligaments. Notably, no Osterix^+^ osteoprogenitors were found in osteoclast resorption areas, indicating uncoupled bone resorption and formation. Even at the late and maturation stages, the uncoupled osteoclast resorption in bony interspinous ligament activates TGF-β to induce the progression of ossification in AS patients. Osteoclast resorption of calcified cartilage-initiated ossification in the progression of AS is a similar pathologic process of acquired heterotopic ossification (HO). Our finding of cartilage formation in the ligaments of AS patients revealed that the pathogenesis of spinal fusion is a process of HO and explained why anti-inflammatory treatments do not slow ankylosing once there is new bone formation in spinal soft tissues. Thus, inhibition of HO formation, such as osteoclast activity, cartilage formation, or TGF-β activity could be a potential therapy for AS.

## Introduction

Ankylosing spondylitis (AS) is an autoimmune disease that mainly affects the spine and sacroiliac joint with a progressive increase in the stiffness of soft tissues, including ligaments, as a common type of spondyloarthritis. The prevalence rates of AS are estimated to range from 0.1% to 0.32% worldwide. Affecting nearly 1.3–1.6 million patients in Europe and 4.6–5.0 million patients in Asia, this disease more commonly occurs in North America with a prevalence of 31.9 per 10 000 of the population.^1^ The inflammation often leads to calcification and bone formation with destructive bone lesions, resulting in spinal fusion, loss of flexibility, and chronic back pain. Significant progress in understanding the cause of this autoimmune disease and developing treatment for inflammation has been achieved in the last decade.^2^ Immune cells and innate cytokines, particularly the human leukocyte antigen (HLA)‑B27 and interleukin‑23/17 axis, are crucial in the pathogenesis of AS. Since its discovery, HLA-B27 has been considered the major genetic risk factor for AS.^3^ Advances in the study of autoimmune diseases and anti-inflammatory treatments have not slowed ankylosis mainly because of the limited understanding of how inflammation induces fusion of axial joints.^2,4^

Treatment with the anti-TNF-α antibody effectively reduces active inflammatory lesions by up to 80% in 6–24 months, as shown by MRI.^5^ This reduction would also be expected to halt the progression of AS; however, no significant reduction in spine ossification and fusion has been reported, not even as significant as NSAID.^6,7^ Indeed, the overexpression of human TNF-α in mice is associated with the development of sacroiliitis.^8^ Radiographic progression in AS patients treated with infliximab suggests that bony structural progression is independent of TNF-α and varies from other inflammatory rheumatic diseases such as rheumatoid arthritis and psoriatic arthritis.^9^ MRI data show that new bone formation in syndesmophytes is three times more likely to occur in the active inflammation area than in the area with no active inflammation, and that syndesmophytes occur at sites with previous inflammation but not persistent inflammation.^10^ Long-term anti-TNFα therapy using different anti-TNF-α agents has improved spinal inflammation in AS patients but no signs of inhibition of structural damage progression have been found.^11–14^ Thus, no simple relationship is identified between the inhibition of inflammation and bony progression, suggesting that inflammation does not directly induce new bone formation.

In fact, the development of ankylosis is uncoupled from inflammation.^15^ An in vivo experiment using well-studied DBA/1 mice demonstrated the uncoupling of inflammation and joint remodeling in spondylarthritis.^16^ Patients who had undergone treatment for anti-inflammation still developed syndesmophytes at sites where no inflammation had been detected by MRI.^17^ Moreover, MRI detection of inflammation in a vertebral unit only slightly increased the likelihood of new syndesmophyte formation and did not affect the growth of existing syndesmophytes.^18^ Inflammation seems to be a precondition of bony formation in AS.

A systematic study of specimens from AS patients demonstrated the presence of a woven bone within the sacroiliac ligament,^18^ suggesting that AS is not merely enthesitis caused bone formation. In addition to excessive bone formation, osteoporosis in AS patients has a prevalence rate of 9.5%–40%.^19–23^ The level of osteoclastic foci in the subchondral bone marrow and the number of osteoclasts at trabecular bone were significant higher than non-AS controls,^24,25^ and the level of osteoclastic bone resorption is not related to the inflammatory activity.^25^ Bisphosphonates are widely known drugs that not only inhibit osteoclasts but also improve osteoporotic bone density and prevent fractures.^26^ AS patients treated with bisphosphonate achieve similar reductions in disease activity, which take the BASDAI and other clinical index as efficacy measure, compared with anti-inflammatory therapy and benefits to bone mineral density.^27^ Treatment with bisphosphonates either improves the clinical index or ameliorated radiological progression in AS patients,^28–31^ regardless of whether a short-term^28^ or intermittent treatment was adopted.^29^ Therefore, bony formation in AS not only originates from enthesitis; osteoclasts also play an important role in structural disease progression although the underlying mechanism has yet to be elucidated.

Acquired heterotopic ossification (HO) is a painful and debilitating disease characterized by extraskeletal bone formation after injury. We reported that transforming growth factor-beta (TGF-β) initiates and promotes HO during four stages: (1) inflammation, (2) chondrogenesis, (3) osteogenesis, and (4) maturation.^32^ Chondrocyte differentiation and cartilage formation is an intermediate phase before HO. HO is acquired in most instances, but HO is also detected in some rare genetic diseases such as fibrodysplasia ossificans progressive (FOP)^33^ and progressive osseous heteroplasia.^34^ In FOP, HO also occurs in a endochondral ossification way that cartilage formation is the initiator of subsequent ossification.^35^ Notably, osteoclast resorption of calcified cartilage activates excessive active TGF-β that recruits mesenchymal stromal/progenitor cells in the HO microenvironment for ectopic bone formation.^32,36,37^ Even at the maturation stage, active TGF-β is continuously freed by osteoclastic resorption, driving the progression of HO. Interestingly, The serum level of TGF-β was significantly elevated in AS patients and remained high regardless of disease activity.^38^ Anti-TNF-α treatment improves the functional and disease activity of AS with no effect on the serum level of active TGF-β.^38,39^ An immunohistological study of sacroiliac joint biopsies also revealed that patients with advanced AS had high serum levels of active TGF-β.^40^ The pathogenesis of acquired HO characterized in our previous study suggests that spinal fusion could involve a similar pathological process of acquired HO.^32,36^

In the current study, we found chondrocyte differentiation and cartilage formation in the spinal ligaments of patients with early-stage AS. Numerous giant osteoclasts were observed on the bony surface with elevated active TGF-β, which promoted the recruitment of Osterix^+^ osteoprogenitors for ossification of spinal fusion. The results demonstrate that AS progression also undergoes same stages: inflammation, chondrogenesis, osteogenesis, and maturation as pathogenesis of acquired HO. Therefore, inhibition of cartilage formation and osteoclast resorption activity can be an effective therapy for AS.

## Results

### Progression of ossification of spinal ligaments and hip joint ligaments of AS patients

**Fig. 1:**
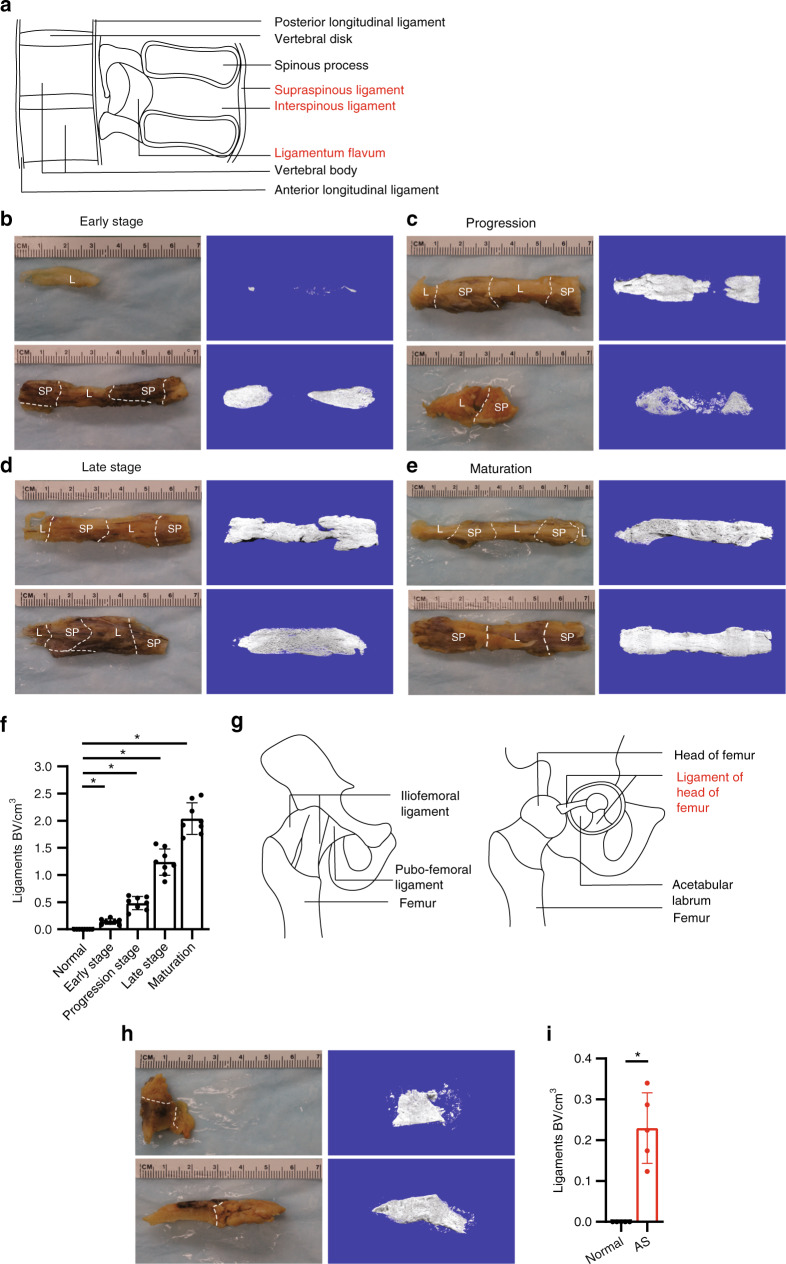
μCT scan of calcified ligaments in AS patients. **a** Illustration of spinal ligaments. Unaided views of spinous process specimens (interspinous ligament with supraspinous ligament and/or ligamentum flavum) and their μCT scans showing **b** limited calcification, **c** partial calcification, **d** complete calcification of ligaments, and **e** mature bony ligaments. **f** Quantification of heterotopic bone volume (BV) in ligaments of the spinous process. **g** Illustration of hip joint ligaments. **h** Unaided view of calcified ligaments of a hip joint specimen and its μCT scan. **i** Quantification of heterotopic bone volume in ligaments of the hip joint in normal and ankylosing spondylitis (AS) specimens. L, ligament; SP, spinous process; AL, acetabular labrum; FH, femoral head

We collected 50 surgery specimens of AS patients from fused spine or hip joints. These specimens were systemically scanned and analyzed by microcomputerized tomography (μCT). The images indicated that the specimens obtained from patients included the interspinous ligament with the supraspinous ligament, with/without the ligamentum flavum, and with/without the posterior longitudinal ligament (Fig. 1a). We observed AS progression of ossification in the spinal ligaments, particularly in the interspinous ligaments because the interspinous ligaments were retained in all specimens we collected. Ossification was visible (without aid) at different stages: the early stage (onset of ossification, Fig. 1b), the progression stage (partial ossification, Fig. 1c), the middle–late stage (incomplete ossification, Fig. 1d), and the mature stage (complete ossification, Fig. 1e). μCT analysis of these four stages of interspinous ligaments also revealed that bone volumes were increased as the condition progressed from the early stage to the maturation stage (Fig. 1f); meanwhile, no ossification within normal ligaments was observed. We also analyzed specimens obtained from AS patients with fused hip joints. The illustration indicates that the specimens from the hip joints of the patients included ligaments of femur head, which was attached to either the head of the femur or the acetabular labrum (Fig. 1g). We observed ossification in ligaments of the hip joints, and μCT analysis of these ligaments also indicated the presence of ossification within these ligaments, whereas no ossification was evident in normal ligaments. Three-dimensional reconstruction of the μCT scan (Fig. 1b–e, h) demonstrated that before ligaments became complete bony structures, most interspinous ligaments in the specimen underwent ossification. Thus, we systematically determined whether spinal fusion in the soft tissue between vertebral bodies such as interspinous ligaments, supraspinous ligaments, and the fusion of hip joints is a process of HO.

### Early stage: inflammation marked by increased CD68^+^ macrophages and TGF-ß activity

**Fig. 2:**
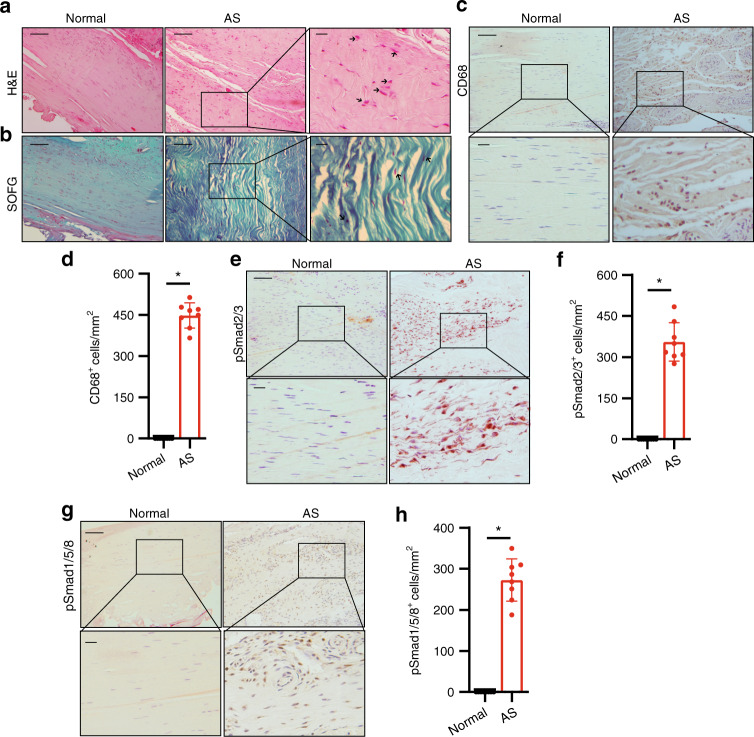
Elevated TGF-β levels in the early inflammatory stage of AS. **a** Hematoxylin and eosin (H&E) staining and **b** Safranin O–Fast Green (SOFG) staining of normal and inflamed interspinous ligaments. In the AS group, the right panels show magnified views of the boxed area in the left panels. Scale bar: 100 μm (two panels on the right); 25 μm (left panel). **c** Immunostaining and **d** quantitative analysis of CD68-positive cells (brown) in the normal and inflamed interspinous ligaments (sagittal view). The bottom panels show magnified views of the boxed area in the top panels. Scale bar: 100 μm (top panels); 25 μm (bottom panels). **e** Immunostaining and **f** quantitative analysis of pSmad2/3-positive cells (brown) in the normal and inflamed interspinous ligaments (sagittal view) in normal ligaments and inflammatory ligaments. The bottom panels show magnified views of the boxed area in the top panels. Scale bar: 100 μm (top panels); 25 μm (bottom panels). **g** Immunostaining and **h** quantitative analysis of pSmad1/5/8-positive cells (brown) in the normal and inflamed interspinous ligaments (sagittal view). The bottom panels show magnified views of the boxed area in the top panels. Scale bar: 100 μm (top panels); 25 μm (bottom panels)

We first performed immunohistology analysis of the spinal ligament specimens from the early (inflammation) stage of AS. Sagittal sections were prepared from the embedded interspinous ligament specimens after decalcification. Hematoxylin and eosin (H&E) staining of noncalcified area showed that the fibers were disorganized and infiltrated with more cells than those in the normal control. The shape of ligament cells changed from fusiform to circular (as indicated by a black arrow) (Fig. 2a). Safranin O–Fast Green (SOFG) staining of sagittal sections in the noncalcified area of AS interspinous ligaments showed no calcification and cartilage formation relative to those of normal control (Fig. 2b). However, immunostaining indicated a significant increase in CD68^+^ macrophages in the AS interspinous ligament (Fig. 2c, d), indicating the presence of inflammation when no calcification occurred. Notably, immunostaining for phosphorylated Smad2/3 (pSmad2/3), a downstream signaling transducer of TGF-β, revealed that the pSmad2/3^+^ cells significantly increased in the inflammation area of the ligaments affected by AS (Fig. 2e, f). These results indicate that TGF-β activity was significantly increased at the early inflammatory stage of AS. We also found higher levels of pSmad1/5/8 signaling in the ligaments of AS patients than those in normal controls (Fig. 2g, h), suggesting active bone morphogenetic protein (BMP) signaling.

### Progression stage: chondrogenesis in the interspinous ligaments

**Fig. 3:**
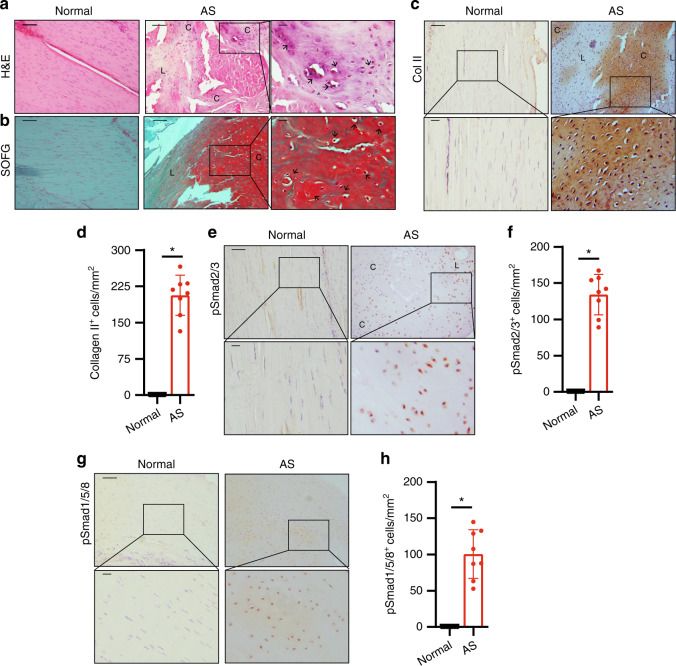
Chondrocyte differentiation and cartilage formation in interspinous ligaments of AS patients before calcification. **a** Hematoxylin and eosin (H&E) staining and **b** Safranin O–Fast Green (SOFG) staining of normal and chondrogenic interspinous ligaments. In the AS group, the right panels show magnified views of the boxed area in the left panels. Scale bar: 100 μm (two panels on the right side); 25 μm (left panel). **c** Immunostaining and **d** quantitative analysis of collagen II-positive cells (brown) in the normal and chondrogenic interspinous ligaments (sagittal view). The bottom panels show a magnified view of the boxed area in the top panels. Scale bar: 100 μm (top panel); 25 μm (bottom panel). **e** Immunostaining and **f** quantitative analysis of pSmad2/3-positive cells (brown) in the normal and chondrogenic interspinous ligaments (sagittal view). The bottom panels show magnified views of the boxed area in the top panels. Scale bar: 100 μm (top panel); 25 μm (bottom panel). **g** Immunostaining and **h** quantitative analysis of pSmad1/5/8-positive cells (brown) in the normal and inflammatory interspinous ligaments (sagittal view). The bottom panels show magnified views of the boxed area in the top panels. Scale bar: 100 μm (top panels); 25 μm (bottom panels). C, cartilage; L, ligament

Increased TGF-b activity promotes chondrogenesis as an essential stage of HO. To examine whether spinal fusion undergoes cartilage formation, we performed histological analysis of AS interspinous ligaments with limited calcification areas at the progression stage as determined by μCT. H&E staining of sagittal sections of AS interspinous ligaments showed the morphologic features of cartilage formation in the ligaments (Fig. 3a, denoted by a black arrow). SOFG staining of the sagittal sections of interspinous ligaments in AS confirmed cartilage formation with chondrocytes in the cartilage lacuna (Fig. 3b). Moreover, immunostaining for collagen II (Col II) in chondrocytes demonstrates that Col II^+^ cells were significantly increased in the interspinous ligaments of AS patients relative to those from the normal control, indicating chondrocyte differentiation and cartilage formation (Fig. 3c, d). Immunostaining for pSmad2/3 showed that the pSmad2/3^+^ cells significantly increased in the interspinous ligaments, particularly at chondrogenesis sites, of AS patients (Fig. 3e, f). These results reveal that spinal soft tissues underwent chondrogenesis in the interspinous ligaments of AS patients before calcification. During this stage, the pSmad1/5/8 intensities were also higher in the ligaments of AS patients relative to those from the normal control but not as much as those of pSmad2/3 signaling (Fig. 3g, h).

### Middle–late stage: endochondral ossification in the bony interspinous ligaments of the fused spine

**Fig. 4:**
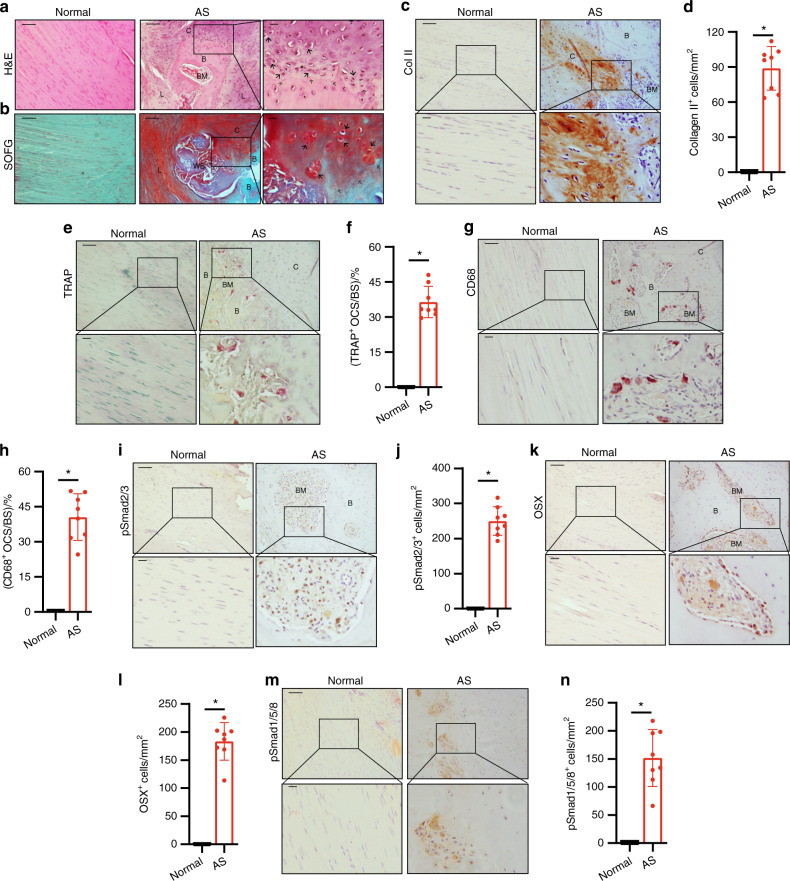
Progression of endochondral heterotopic ossification in AS patients. **a** Hematoxylin and eosin (H&E) staining and **b** Safranin O–Fast Green (SOFG) staining of normal interspinous ligaments and endochondral-ossified interspinous ligaments. In the AS group, the right panels show magnified views of the boxed area in the left panels. Scale bar: 100 μm (two panels on the right side); 25 μm (left panel). **c** Immunostaining and **d** quantitative analysis of collagen II-positive cells (brown) in the normal and chondrogenic interspinous ligaments (sagittal view). The bottom panels show magnified views of the boxed area in the top panels. Scale bar: 100 μm (top panels); 25 μm (bottom panels). **e** Tartrate-resistant acid phosphatase (TRAP)-positive cells (red) and **f** quantitative analysis of TRAP-positive osteoclast (red) surface (OCS) per bone surface (BS). The bottom panels show magnified views of the boxed area in the top panels. Scale bar: 100 μm (top panel); 25 μm (bottom panel). **g** Immunostaining and **h** quantitative analysis of the number of CD68-positive osteoclast (brown) surface (OCS) per bone surface (BS). The bottom panels show magnified views of the boxed area in the top panels. Scale bar: 100 μm (top panel); 25 μm (bottom panel). **i** Immunostaining and **j** quantitative analysis of pSmad2/3-positive cells (brown) in the normal ligaments and endochondral-ossified interspinous ligaments (sagittal view). The bottom panels show magnified views of the boxed area in the top panels. Scale bar: 100 μm (top panel); 25 μm (bottom panel). **k** Immunostaining and **l** quantitative analysis of Osterix-positive cells (brown) in the normal and endochondral-ossified interspinous ligaments (sagittal view). The bottom panels show magnified views of the boxed area in the top panels. Scale bar: 100 μm (top panel); 25 μm (bottom panel). **m** Immunostaining and **n** quantitative analysis of pSmad1/5/8-positive cells (brown) in the normal and inflammatory interspinous ligaments (sagittal view). The bottom panels show magnified views of the boxed area in the top panels. Scale bar: 100 μm (top panels); 25 μm (bottom panels). B, bone; BM, bone marrow; C, cartilage

Chondrogenesis in the interspinous ligaments in AS suggests that spinal fusion in AS is an HO process. We then examined whether chondrogenesis in the progression stage leads to endochondral ossification of HO. Interspinous ligaments at the middle–late stage of AS were embedded and sections were prepared. H&E staining of the sagittal sections of the calcified area in the interspinous ligaments affected by AS revealed the formation of a new bone with marrow surrounded by thick layers of cartilage (Fig. 4a), indicating endochondral ossification. SOFG staining confirmed that the calcified green-stained area was surrounded by Safranin O red cartilage with chondrocytes and cartilage lacuna and that the boundary between the cartilage and the bone was distinctly visible (Fig. 4b, denoted by a black hollow arrow). Moreover, immunostaining for collagen II revealed that a large number of collagen II-positive cells were at the cartilage sites adjacent to the bone and marrow (Fig. 4c, d). As a process of endochondral bone formation in HO, resorption of calcified cartilage by osteoclasts activates TGF-β to recruit osteoprogenitors. Tartrate-resistant acid phosphatase (TRAP) staining for osteoclasts revealed the presence of a large number of TRAP-positive osteoclasts on the bone surface in the marrow resorption cavity (Fig. 4e, f). Same as TRAP staining, CD68 immunostaining showed a large number of CD68^+^ osteoclasts on the bone surface (Fig. 4g, h). Meanwhile, pSmad2/3 Immunostaining demonstrated that the pSmad2/3 level in the bone marrow significantly increased relative to that in the normal control (Fig. 4i, j), suggesting TGF-b activation during osteoclast resorption. The release of active TGF-b from osteoclast resorption recruits osteoprogenitors for new bone formation. Osterix (OSX) immunostaining showed that Osterix^+^ osteoprogenitors were lined on the bone surface (Fig. 4k, l). No OSX^+^ osteoprogenitors and TRAP-positive osteoclasts were found in the same marrow areas, indicating the uncoupling of bone resorption and bone formation. The results generally reveal that endochondral bone formation proceeded on calcified cartilage during AS progression. At this stage, pSmad1/5/8 signaling was also activated in the ligaments of AS patients; however, similar to the chondrogenesis stage, its activity was not significantly elevated as pSmad2/3 signaling (Fig. 4m, n).

### Mature stage: uncoupled osteoclast resorption of fused bony ligaments drives progression of AS

**Fig. 5:**
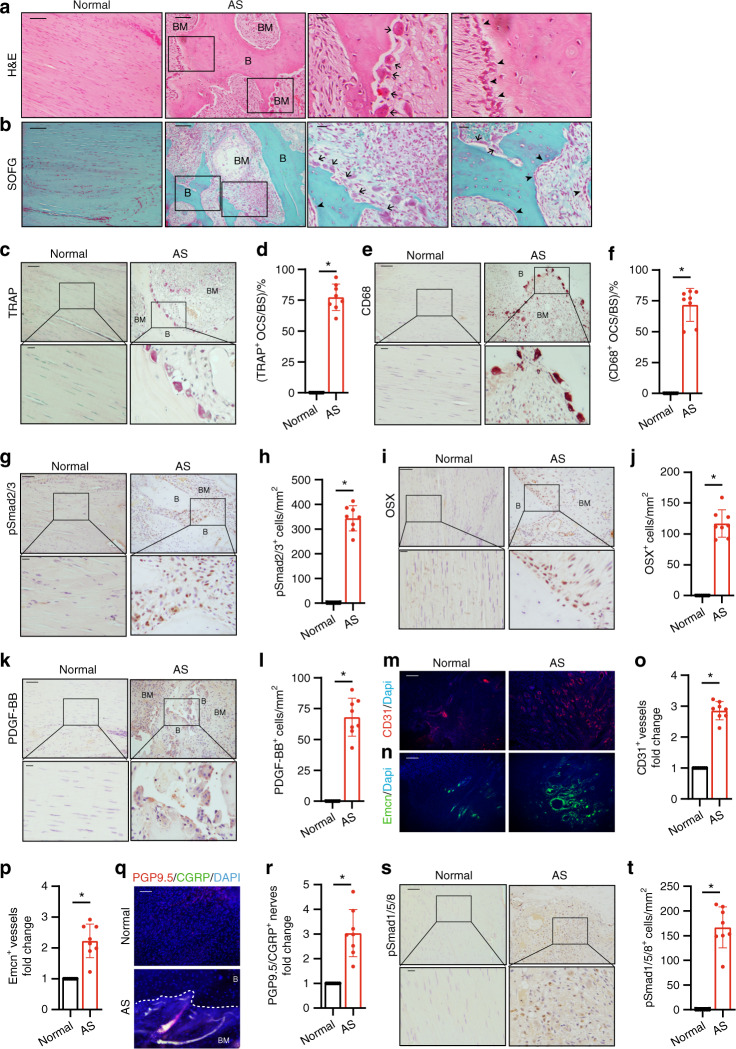
Osteoclast resorption of bony interspinous ligaments releases active TGF-β to drive the progression of ossification in AS patients. **a** Hematoxylin and eosin (H&E) staining and **b** Safranin O–Fast Green staining of normal and HO-formed interspinous ligaments. In the AS group, the right panels show magnified views of the boxed area in the left panels. Scale bar: 100 μm (two panels on the right); 25 μm (two panels on the left). **c** Tartrate-resistant acid phosphatase (TRAP)-positive cells (red) and **d** quantitative analysis of the TRAP-positive osteoclast surface (OCS) per bone surface. The bottom panels show magnified views of the boxed area in the top panels. Scale bar: 100 μm (top panel); 25 μm (bottom panel). **e** Immunostaining and **f** quantitative analysis of CD68-positive cells (brown) in the normal and HO-formed interspinous ligaments (sagittal view). The bottom panels show magnified views of the boxed area in the top panels. Scale bar: 100 μm (top panel); 25 μm (bottom panel). **g** Immunostaining and **h** quantitative analysis of pSmad2/3-positive cells in the normal and HO-formed interspinous ligaments (sagittal view). The bottom panels show magnified views of the boxed area in the top panels. Scale bar: 100 μm (top panel); 25 μm (bottom panel). **i** Immunostaining and **j** quantitative analysis of Osterix-positive cells (brown) in the normal and HO-formed interspinous ligaments (sagittal view). The bottom panels show magnified views of the boxed area in the top panels. Scale bar: 100 μm (top panel); 25 μm (bottom panel). **k** Immunostaining and **l** quantitative analysis of PDGF-BB-positive cells in the normal and HO-formed interspinous ligaments (sagittal view). The bottom panels show magnified views of the boxed area in the top panels. Scale bar: 100 μm (top panels); 25 μm (bottom panels). **m** CD31-positive (red) cells, **n** EMCN-positive (green) cells and quantification of the fold change of **o** CD31-positive vessels, **p** EMCN-positive vessels in the normal and HO-formed interspinous ligaments (sagittal view). **q** PGP9.5-positive (red) cells and CGRP-positive (green) cells and quantification of the fold change of **r** PGP9.5 and CGRP double positive nerves in the normal and HO-formed interspinous ligaments (sagittal view). **s** Immunostaining and **t** quantitative analysis of pSmad1/5/8-positive cells (brown) in the normal and inflammatory interspinous ligaments (sagittal view). The bottom panels show magnified views of the boxed area in the top panels. Scale bar: 100 μm (top panels); 25 μm (bottom panels)

We showed that osteoclast resorption activates TGF-β, promoting HO progression even at the middle–late and maturation stages. Therefore, we determined whether the fused spine (at the maturation stage) was still actively remodeled by osteoclasts for AS progression and pain sensory innervation. The presence of bony interspinous ligaments at the mature stage was confirmed by μCT, and the ligaments were embedded in preparation for sectioning (Fig. 1e). H&E, Safranin O, and fast green staining of mature AS ligaments revealed numerous giant osteoclasts on the bone surface and green-stained bone formation (Fig. 5a, b). TRAP staining confirmed that a large number of osteoclasts were formed on the bone surface (Fig. 5c, d). These particular osteoclasts were considerably larger than those observed at the progression stage. CD68^+^ immunostaining further showed the formation of giant osteoclasts on the bone surface (Fig. 5e, f). Moreover, immunostaining revealed high pSmad2/3 levels in the in the marrow cavity (Fig. 5g, h) and a significant increase in OSX^+^ osteoprogenitors on the bone surface (Fig. 5i, j). In mice, osteoclastic bone remodeling is often coupled with angiogenesis, specifically type-H blood vessel formation through the secretion of platelet-derived growth factor BB (PDGF-BB). Immunostaining showed that the PDGF-BB level was significantly higher in the marrow cavity of mature AS ligaments relative to those of the control (Fig. 5k, l). Moreover, CD31 and Endomucin (EMCN) immunostaining revealed a significant increase in blood vessel formation (Fig. 5m–p). Protein Gene Product 9.5 and calcitonin gene-related peptide positive (CGRP) co-staining revealed dramatically nerve fibers ingrowth at HO site (Fig. 5q, r). These results indicate that osteoclastic remodeling of the fused spine activates TGF-β in the coupling of new bone formation and angiogenesis to promote AS progression, accompany with innervation. The results also suggest that the formation of giant osteoclasts was still active remodeling of the bony spine to drive the progression of AS at the maturation stage. BMP signaling was also explored by evaluating pSmad1/5/8 signaling, which exhibited high intensity in the marrow of the newly formed ectopic bone (Fig. 5s, t).

**Fig. 6:**
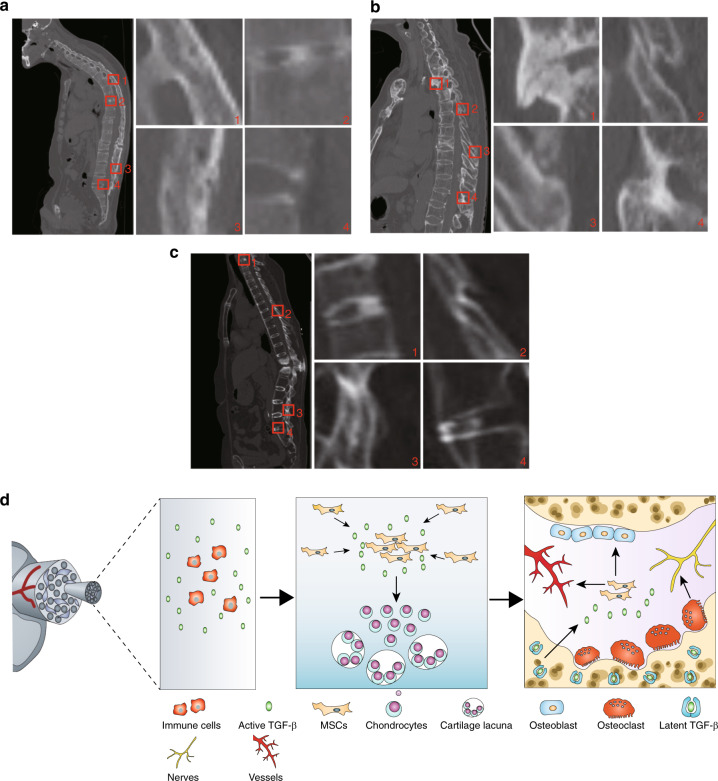
Ossification in spine soft tissues by CT in AS patients. **a** Male, aged 45 years. 1 Supraspinous ligament and Interspinous ligament. 2 Vertebral disk. 3 Supraspinous ligaments. 4 Posterior longitudinal ligaments. **b** Male, aged 37 years. 1 Anterior longitudinal ligament. 2 Interspinous ligament. 3 Interspinous ligament and supraspinous ligament. 4 Interspinous ligament and ligamentum flavum. **c** Female, aged 33 years. 1 Vertebral disk. 2 Interspinous ligament and Ligamentum flavum. 3 Interspinous ligament. 4 Anterior longitudinal ligaments. The right four panels show magnified views of the red boxed area in the left panels. **d** Hypothetical diagram of pathological progression of AS. Excessive TGF-β is produced by abundant immune cells due to autoimmune disease, then TGF-β recruits MSCs results in chondrocytes proliferation, hypertrophy, and calcification. Numbers of giant osteoclasts keep resorbing calcified cartilage and freeing TGF-β from lap, continuous high level of TGF-β recruits MSCs for vessels formation and osteoblasts differentiation, osteoclasts also induce the innervation process

Finally, we evaluated ectopic bone formation in different soft tissues of the spine in AS patients, as determined by CT. CT scans of AS patients with different ages and genders revealed the ectopic bone formation at the vertebral disk and soft tissues of the spine in the supraspinous ligament, interspinous ligament, posterior longitudinal ligament, anterior longitudinal ligament, interspinous ligament, and ligamentum flavum (Fig. 6a–c). The observation suggests that ectopic bone formation occurs in different soft tissues of the AS spine, likely through the HO process.

## Discussion

New bone formation along the axial skeleton is the hallmark of post-inflammation AS, beginning at the sacroiliac joints. Anti-inflammation therapies such as the anti-TNF-α antibody, effectively reduce inflammation but are unable to slow stiffness and fusion in the spine of AS patients. Using immunohistostaining, we found that cartilage formation in the spinal ligaments in AS patients and chondrocytes was clearly detected in the cartilage lacuna. Moreover, col II staining verified the presence of numerous col II^+^ chondrocytes during chondrogenesis within the ligaments. Notably, SOFG staining revealed that calcified tissue was adjacent to cartilage, indicating endochondral ossification in the spine of AS patients. The finding of chondrogenesis in AS patients shows that cartilage formation acts as an intermediate stage to mediate ossification progression from an inflammatory microenvironment. Indeed, the level of active TGF-β significantly increased at the inflammation and progression stages in the spine of AS patients. TGF-β has a broad spectrum of functions, including inflammation, cell migration, chondrogenesis, angiogenesis, epithelial–mesenchymal transition, and remodeling of the new extracellular matrix.^32,41–43^ TGF-β plays an essential role in chondrocyte differentiation, cartilage formation, and bone remodeling.^40,44,45^ We have shown that an inflammatory environment with high levels of active TGF-β provides the conditions needed for cartilage formation as an intermediate phase leading to endochondral ossification in acquired HO.^32^ Thus, cartilage formation in AS patients shows that spinal fusion involves a process similar to that of acquired HO.

Acquired HO has four stages as shown in our previous study: inflammation, chondrogenesis, osteogenesis, and maturation.^32^ Chondrogenesis leads to osteogenesis in HO progression. Analysis of incomplete ossification of ligaments in AS patients revealed that endochondral ossification occurred within the cartilage, in which chondrocytes hypertrophied and then calcified. Notably, TRAP staining showed many TRAP^+^ osteoclasts on the surface of bone cavities. Invasion and aberrant resorption by these giant CD68^+^ osteoclasts generated bone cavities as in the function of marrow.^46,47^ High levels of active TGF-β were detected in the marrow cavity as evidenced by a large number of pSmad2/3^+^ cells. TGF-β activated by osteoclast bone resorption recruits MSCs in coupling with new bone formation.^40,41^ Indeed, abundant OSX^+^ progenitors were detected in the marrow cavity; however, they were not located at osteoclast-resorptive sites but rather dispersed in areas with active bone formation. This occurrence characterizes typical uncoupled bone resorption and formation as observed in HO.^32^ Bone formation is always associated with angiogenesis, such as type-H blood vessel formation in mice.^48–50^ During remodeling, TRAP^+^ osteoclastic lineage cells secrete PDGF-BB for angiogenesis and Netrin-1 for sensory innervation.^48,51,52^ Highly abundant blood vessels were observed during HO progression. In AS patients, highly elevated levels of PDGF-BB as the most upstream regulator for blood vessel formation were found in the marrow cavity. Transformation of vascular endothelial cells into multipotent stem-like cells in patients with FOP contributes to heterotopic cartilage and bone formation;^53^ thus, these over-growth blood vessels may facilitate more ectopic bone formation. Back pain is one of the main clinical manifestations in AS^2^, it may be caused by sensory innervation at pathologic sites which is similar with low back pain.^54^ CGRP^+^ nerve fibers were detected in the bony ligaments of AS patients. Giant osteoclasts are likely responsible for the PDGF-BB-induced blood vessel formation and CGRP^+^ nerve innervation.^48,51,52^

More than 100 clinical trials have been conducted for AS, but no effective treatment has been determined for the reduction of both disease activity and structural progression. NSAIDs are the first-line treatment for active AS, providing relief for inflammatory back pain and improving functional activity in AS patients.^55^ However, continuous use of NSAIDs did not further delay AS in any clinical aspect.^56^ The TNF-α antibody or inhibitors can effectively reduce inflammation in AS patients, but no significant effect on halting osteogenesis in the progression of spinal fusion. In addition, TNF-α inhibitors are not equally effective in all patients; they appear to be most effective in males, patients with a relatively short disease duration, patients positive for HLA-B27, those aged ≤40 years, those without enthesitis, those with good functional status, and those with a high C-reactive protein level.^57,58^ Therefore, neither NSAID treatment nor TNF-α inhibitors could halt the progression of structural changes in AS.^6,7,10–12^ Notably, AS clinical trials with bisphosphonates showed greater efficiency in improving the symptoms and changes in the spinal structure, compared with the TNF-α antibody therapy.^27^

The findings on AS in the current study explain the results of preceding clinical trials. The progression of AS involves process of acquired HO and autoimmune inflammation generates an environment supporting cartilage formation to initiate ossification. It appears that inhibition of inflammation does not effectively halt ossification. Particularly, once cartilage has been formed, sites of ossification are continuously remodeled by osteoclast bone resorption, and inflammatory microenvironment will be no longer required.^42,59^ During the maturation stage, interspinous ligaments become completely bony structures, as shown by μCT scans. However, histological staining demonstrates that a large number of giant osteoclasts remain, with high levels of active TGF-β in the remodeling marrow of bony interspinous ligaments. Aberrant osteoclastic bone resorption drives the progression of ossification. This occurrence explains how the inhibition of osteoclast activity by bisphosphonates can slow new bone formation. Our findings also suggest that inhibition of the components of HO such as cartilage formation, osteoclastic resorption, or TGF-β activation can be an effective therapy for AS.

## Materials and methods

### Human subjects

The study was approved by the Johns Hopkins University and Peking Union Medical College Hospital review board. All pathology tissues were obtained with written informed consent from individuals. Pathological specimens of spine were collected from 32 patients with AS (29 males and 3 females with no fibrous dysplasia of bone or other blastic bone metastases, nonsmoking, aged 26–64 years, with disease duration of 10–40 years) who met the 1984 modified New York criteria after spinal wedge osteotomy. HO was first accessed using the CT scan results of each patient before enrollment and identified using the μCT scan after collection. Healthy specimens of spine were collected from 8 patients (5 males and 3 females with no fibrous dysplasia or other blastic bone metastases, nonsmoking, aged 23–65 years) after surgical treatment of spinal fracture. Pathological specimens of hip joint were collected from 5 patients with AS (5 males with no fibrous dysplasia of bone or other blastic bone metastases, nonsmoking, aged 33–64 years, with disease duration of 14–37 years) who met the 1984 modified New York criteria after total hip joint replacement. Healthy specimens of hip joint were collected from 5 patients (5 males with no fibrous dysplasia or other blastic bone metastases, nonsmoking, aged 55–69 years) after surgical treatment of fracture. The obtained specimens were defined based on the μCT results and included the following: the hip joint (the femoral head with ligaments and acetabular labrum with ligaments, spinous process with the interspinous ligament, supraspinous ligament and/or ligamentum flavum. We determined the following stages of AS through μCT analysis of the spinal ligaments: early stage (disease duration, 10–18 years; 6 men and 2 women), progression stage (disease duration, 10–21 years; 7 men and 1 woman), late stage (disease duration,15–27 years; 8 men), and mature stage (disease duration, 20–40 years; 8 men).

### MicroCT analysis

Human tissues were fixed overnight in 10% formalin and analyzed by high-resolution μCT (Skyscan1172, Skyscan1272, Bruker microCT, Kontich, Belgium) and included the hip joint (the femoral head with ligaments and the acetabular labrum with ligaments), the spinous process with the interspinous ligament, and the supraspinous ligament and/or the ligamentum flavum from both healthy patients and AS patients. The scanner was set at a voltage of 60 kV and a resolution of 9 μm per pixel. Images of perfusion computed tomography (PCT) were used to perform three-dimensional (3D) histomorphometric analyses. The region of interest was defined to cover the whole PCT compartment (the femoral head or acetabular labrum with ligaments, single spinous process or joint spinous process with the interspinous ligament, supraspinous ligament, and/or ligamentum flavum). The images were reconstructed with NRecon v1.6 software (Bioz, Inc., Palo Alto, CA, USA), analyzed by CTAn v1.9 software (Bruker microCT), and visualized using the 3D model visualization software CTVol v2.0 (Bruker microCT).

### Histochemistry

The blocks were sectioned at 4 μm by using a paraffin microtome (for paraffin blocks). We processed bone sections with a thickness of 4 μm for H&E staining as well as Safranin O (S2255, Sigma-Aldrich, St. Louis, MO, USA) and Fast Green (F7252, Sigma-Aldrich, St. Louis, MO, USA) staining. We performed all staining using paraffin-embedded sections. The sections were dewaxed and then washed three times with phosphate-buffered saline (PBS). The sections were stained with hematoxylin (H9627, Sigma-Aldrich, St. Louis, MO, USA) for 5 min, rinsed in running tap water for 10 s, differentiated with 0.3% acid alcohol for 10 s, and rinsed again in running tap water for 1 min, followed by counterstaining with Eosin Y (7111, Thermo-Fisher Scientific, Inc., Walkersville, MD, USA) for 1 min. For SOFG staining, the sections were counterstained with Fast Green for 5 min and then differentiated with 1% acetic acid for 10 s. Subsequently, the sections were counterstained with Safranin O for 5 min. TRAP staining was conducted in accordance with the protocol provided by the manufacturer (387A-1KT, Sigma-Aldrich, St. Louis, MO, USA), followed by counterstaining with Methyl Green (M884, Sigma-Aldrich, St. Louis, MO, USA).

### Immunohistochemistry

The blocks were sectioned at 4 or 60 μm (for CD31 and EMCN immunofluorescence staining) intervals by using a Microm cryostat (for frozen blocks, Fisher Scientific) or a paraffin microtome (for paraffin blocks). For immunohistochemical staining, paraffin sections that had been dewaxed were heated to 99 °C for 15 min in Target Retrieval Solution (S1699, Dako, Agilent Technologies, Santa Clara, CA, USA) for antigen retrieval and then for 30 min at room temperature. The sections were subsequently rehydrated. The tissue sections were washed three times with PBS, incubated with a blocking solution for 1 h, and incubated with primary antibodies to human pSmad2/3 (sc-11769, 1:50, Santa Cruz Biotechnology Inc.), human CD68 (MA5–13324, 1:100, Invitrogen, Carlsbad, CA, USA), human collagen II (MA5–12789,1:100, Invitrogen, Carlsbad, CA, USA), human Osterix (ab22552, 1:100, Abcam, Cambridge, MA, USA), human PDGF-BB (ab21234, 1:50, Abcam, Cambridge, MA, USA) overnight at 4 °C in a humidified chamber, followed by incubation for 1 h at room temperature. The sections were washed three times with Tris-buffered saline. We incubated the slides with secondary antibodies in a blocking solution for 1 h at room temperature and used chromogenic substrates (K3468, Dako, Agilent Technologies) to detect the immunoactivity. We ultimately counterstained the slides with hematoxylin (H9627, Sigma-Aldrich, St. Louis, MO, USA). For immunofluorescence staining, we incubated the slides at 37 °C for 30 min and washed the sections three times with PBS. The sections were incubated with a blocking solution for 1 h and then with primary antibodies to human CD31 (ab28364, 1:100, Abcam, Cambridge, MA, USA), human Endomucin (V.7C7, sc-65495, 1:50, Santa Cruz Biotechnology Inc.) overnight at 4 °C in a humidified chamber, followed by incubation for 1 h at room temperature. The sections were washed three times with Tris-buffered saline. We then used secondary antibodies conjugated with fluorescence at room temperature for 1 h while avoiding light. The sections were mounted in ProLong Gold Mounting Reagent with DAPI (P36935, Life Technologies). We used isotype-matched controls, such as polyclonal rabbit IgG (AB-105-C, R&D Systems, Minneapolis, MN, USA), polyclonal goat IgG (AB-108-C, R&D Systems, Minneapolis, MN, USA), and monoclonal rat IgG2A (54447, R&D Systems, Minneapolis, MN, USA) under the same concentrations and conditions as the negative controls.

### Histomorphometry

We processed the imaging samples by using an Olympus DP71 microscope (Olympus Scientific Solutions Americas Inc., Waltham, MA, USA). We analyzed the human specimens according to five sequential sections per stain and used anatomical landmarks to ensure comparability, including the presence of bone marrow and bone matrix. Moreover, serial sagittal sections of HO lesions were obtained. We determined the number of positively stained cells in each of five random visual fields in five sequential sections per specimen for each group and normalized the values to the number per millimeter of the adjacent bone surface (for TRAP staining quantification) or per square millimeter in the HO area. We conducted a quantitative analysis using the OsteoMeasureXP Software (OsteoMetrics, Inc., Decatur, GA, USA). For CD31^+^ and EMCN^+^ vessel quantification, we calculated the areas in red (CD31^+^) and green (EMCN^+^) at the whole HO site of each slide in three sequential sections per specimen for each group and normalized the values to those of the control specimens (set to 1). For chondrocyte quantification, we quantified all cells in the brown (Col II^+^) area and considered them as col II^+^ chondrocytes. Quantifications were performed using the software ImageJ 1.53c (National Institutes of Health, Bethesda, MD, USA).

### Statistics

The data were statistically analyzed using SPSS version 15, software (IBM, Armonk, NY, USA), presented as mean ± SD. Unpaired two-tailed Student’s *t*-test was conducted for the separate comparison of the morphometric analysis of the inflammation, chondrogenesis, osteogenesis, or maturation in healthy patients and AS patients. The level of significance was set at *P* < 0.05. All inclusion/exclusion criteria were established beforehand, and no samples were excluded from the analysis. No statistical method was used to predetermine the sample size. The experiments were randomized. The investigators were not blinded to the allocation during the experiments and outcome assessment.

## Data Availability

All data generated or analyzed during this study are included in this article (and its supplementary information files). Please contact the corresponding author for unique material requests. Some material used in the reported research may require requests to collaborators and agreements with both commercial and non-profit institutions, as specified in the paper. Requests are reviewed by Johns Hopkins University to verify whether the request is subject to any intellectual property or confidentiality obligations. Any material that can be shared will be released via a Material Transfer Agreement.
